# Masked-Volume-Wise PCA and "reference Logan" illustrate similar regional differences in kinetic behavior in human brain PET study using [11C]-PIB

**DOI:** 10.1186/1471-2377-9-2

**Published:** 2009-01-07

**Authors:** Pasha Razifar, Anna Ringheim, Henry Engler, Håkan Hall, Bengt Långström

**Affiliations:** 1 Molecular Imaging & CT Research, GE Healthcare, WI 53188, Waukesha, USA; 2 Uppsala Applied Science Laboratory, GE Healthcare, SE-752 28, Uppsala, Sweden; 3 Uppsala Imanet AB, GE Healthcare, Box 967, SE-751 09, Uppsala, Sweden; 4 Department of Medical Science, Uppsala University, SE-751 85 Uppsala, Sweden; 5 Department of Biochemistry and Organic Chemistry, Uppsala University, SE-751 24 Uppsala, Sweden

## Abstract

**Background:**

Kinetic modeling using reference Logan is commonly used to analyze data obtained from dynamic Positron Emission Tomography (PET) studies on patients with Alzheimer's disease (AD) and healthy volunteers (HVs) using amyloid imaging agent *N*-methyl [^11^C]2-(4'-methylaminophenyl)-6-hydroxy-benzothiazole, [^11^C]-PIB. The aim of the present study was to explore whether results obtained using the newly introduced method, Masked Volume Wise Principal Component Analysis, MVW-PCA, were similar to the results obtained using reference Logan.

**Methods:**

MVW-PCA and reference Logan were performed on dynamic PET images obtained from four Alzheimer's disease (AD) patients on two occasions (baseline and follow-up) and on four healthy volunteers (HVs). Regions of interest (ROIs) of similar sizes were positioned in different parts of the brain in both AD patients and HVs where the difference between AD patients and HVs is largest. Signal-to-noise ratio (SNR) and discrimination power (DP) were calculated for images generated by the different methods and the results were compared both qualitatively and quantitatively.

**Results:**

MVW-PCA generated images that illustrated similar regional binding patterns compared to reference Logan images and with slightly higher quality, enhanced contrast, improved SNR and DP, without being based on modeling assumptions. MVW-PCA also generated additional MVW-PC images by using the whole dataset, which illustrated regions with different and uncorrelated kinetic behaviors of the administered tracer. This additional information might improve the understanding of kinetic behavior of the administered tracer.

**Conclusion:**

MVW-PCA is a potential multivariate method that without modeling assumptions generates high quality images, which illustrated similar regional changes compared to modeling methods such as reference Logan. In addition, MVW-PCA could be used as a new technique, applicable not only on dynamic human brain studies but also on dynamic cardiac studies when using PET.

## Background

Imaging modality such as dynamic Positron Emission Tomography, PET, when using amyloid imaging agent *N*-methyl [^11^C]2-(4'-methylaminophenyl)-6-hydroxy-benzothiazole, [^11^C]-PIB [1], is increasingly used as a diagnostic tool for providing early diagnosis of patients with Alzheimer's disease, AD [2-4]. The tracer principle is based on non-invasive estimation of beta-amyloid (Aβ) plaques in the brain of patients with AD vs. healthy volunteers (HVs). The technique is also valuable when exploring deposition of amyloid in different parts of the brain.

However, images generated using PET contain noise with different magnitude, spatial dependence and correlation, which impairs the visualization and affects the precision in quantification of the generated images. This is due to limitations of the amount of the radioactivity associated with the administered tracer, which is usually short-lived, to different technical limitations and to applied corrections and reconstruction algorithm used [5-7].

To extract quantitative values of a desired physiological information and to study the kinetic behavior of administered tracer in human PET studies, different methods can be used, such as kinetic modeling using e.g. the Patlak [4,8,9] and the Logan plots [3,10]. These models need either the determination of the time course of the unchanged tracer concentration in arterial plasma or the definition of a reference region that is devoid of specific binding. Modeling can also be performed on a pixel-wise basis, generating parametric images. Prior knowledge and modeling experience about the kinetic behavior of the administered tracer in the human brain are needed to find the most appropriate method for a particular tracer. Furthermore, parametric images generally suffer from poor quality and a non-optimized signal-to-noise ratio (SNR). There are also many parameters and variables that need to be correctly considered to obtain proper results when applying these techniques.

In previous studies novel approaches for application of Principal Component Analysis (PCA) on dynamic PET images such as Masked Volume Wise PCA (MVW-PCA), were introduced [5,6]. Images generated using MVW-PCA contained more detailed anatomical information with higher quality and precision as compared with images generated using other methods. We explored the performance of MVW-PCA as a way to both improve detection and visualization of significant changes in tracer kinetics by extracting different components and to enhance the discrimination between pathological and healthy regions in the brain. Several well-known tracers in clinical and research practice were used such as [^11^C]-L-deuterium-deprenyl ([^11^C]-DED), [^11^C]-GR205171 ([^11^C]-GLD), [^11^C]-5-hydroxytryptophan ([^11^C]-HTP), [^11^C]L-dihydroxyphenylalanine ([^11^C]-L-DOPA) and [^11^C]-PIB [5].

It was previously shown that MVW-PCA can be used as a multivariate image analysis technique that without modeling assumptions can extract and separate important kinetic information into different component images, MVW-PC images. There are no correlations between the kinetic information separated in these images [5].

The objectives of the current study were to explore the capabilities of MVW-PCA compared to kinetic modeling using the reference Logan method on the same dataset. The methods were used to extract signals which improve the discrimination between AD patients and HVs in a human brain study using PET and [^11^C]-PIB. In other words, the aim of the study was to explore whether images generated by MVW-PCA illustrate similar regional retention patterns compared to reference Logan images. Another aim was to study and compare these methods with respect to signal extraction based on calculating SNR and discrimination power (DP).

## Methods

### Dynamic PET data

Data were used from four arbitrarily chosen AD patients and four HVs recruited for different studies at Uppsala Imanet, GE Healthcare in collaboration with several groups such as Karolinska University Hospital Huddinge, Stockholm, Sweden. The four AD patients were also recruited in a follow-up (FU) study where they were scanned a second time at Uppsala Imanet, GE Healthcare [4]. Data from both the baseline and FU studies were used in this study. Four HVs were selected randomly from a study performed at Uppsala Imanet in collaboration with Karolinska University Hospital Huddinge, Stockholm and University of Pittsburgh School of Medicine, Pittsburgh, USA [2].

### Data analysis

#### Masked Volume Wise Principal Component Analysis

PCA [11,12] is a data-driven and well-established multivariate analysis technique used to reduce dimensionality of multivariate input datasets such as dynamic PET images. PCA converts the projection of the original images into a new orthogonal coordinate system with lower dimensions in which the new axes explain the variance in the images in decreasing order of importance. This is done by calculating transformation vectors (principal components, i.e. PCs or weight factors), which define the directions of largest variance of the input multi-dimensional dataset in the multidimensional feature space. Each PC is orthogonal to all the others in multi-dimensional space; thus, the first PC (PC1) represents the linear transformation of the original variables which contain the largest variance. The second component, PC2, is the combination that contains the remaining variance as much as possible, orthogonal to the previous one and so on. "PC images" are generated by simply projecting all observations onto the PCs in the new multi-dimensional space. This gives new values along each PC.

If the matrix

*X*^*T *^= [*X*_1_, *X*_2_, *X*_3_, ..., *X*_*p*_]

where *X*^*T *^is transpose of the input matrix *X*, which contains pixel values of a single slice from each frame *i*, (*i *= (1, 2, 3, ..., *p*), as a column vector), has a variance-covariance matrix *S *with eigenvalues

*λ *= ⌊*λ*_1_, *λ*_2_, *λ*_3_, ..., *λ*_*p*_⌋

and corresponding eigenvectors

*e *= ⌊*e*_1_, *e*_2_, *e*_3_, ..., *e*_*p*_⌋

where

*λ*_1 _≥ *λ*_2 _≥ *λ*_3 _≥ ... ≥ *λ*_*p*_*≥ *0

and *p *corresponds to the number of the input column in the matrix *X*. If *q *= *p *in which *q *refers to number of principal component, then the *q*^th ^PC is generated by using Eq. 1:

(1) *Y*_*q *_= *e'X *= *e*_*q*1_*X*_1 _+ *e*_*q*2_*X*_2 _+ *e*_*q*3_*X*_3 _+ ... + *e*_*qp*_*X*_*p*_

The condition *Cov*(*Y*_*q*_, *Y*_*i*_) = 0 where *i *≠ *q *is required, i.e. the components are uncorrelated. Principal components should explain the magnitude of variance in decreasing order. Here each element within the eigenvectors is used as weight-factor for creating images.

In a previous work, MVW-PCA was introduced as a new approach of application of PCA on dynamic PET images using various compounds, among others [^11^C]-PIB [5]. This method is based on using noise prenormalized data that represents whole brain of each time sequence (frame) as a single variable after the background had been removed (masked out) before applying PCA. The result is MVW-PC images, which are separated into different principal components, MVW-PCs. The number of components is equal to the number of PET time frames. In each PET dataset, the whole sequence (all frames) was used to include all information in the dataset. Furthermore, the MVW-PC images can be seen as images representing different kinetic behaviors of the administered tracer and contain more detailed anatomical information with higher precision and quality. They have an improved SNR and visual contrast between the anatomical structures representing both affected and unaffected tissues compared with other methods [13].

However, in this work, only the first three MVW-PCs were explored since higher components contained only noise.

The software used for the application of MVW-PCA on dynamic PET images was developed in-house by one of the authors (PR) using Matlab 7.2 (The Mathworks, Natick, Massachusetts) with installed statistical and image processing toolboxes.

#### Reference Logan graphical analysis

The reference Logan graphical analysis is a regression method, which is appropriate for tracers with reversible kinetics. The method describes the kinetics of the tracer in a receptor-containing region and in a reference region devoid of specific binding, such that after a certain time there is a linear relationship between the reference Logan variables. The linear slope gives directly the distribution volume ratio (DVR), which corresponds to the ratio of the distribution volume (DV) of a receptor-containing region to the DV of a reference region. The DVR is widely used as a model parameter in PET studies since it is a linear function of the receptor availability. Parametric maps, in which the DVR is calculated for each pixel in the PET image, were generated using 25–60 min (last six frames) for 4 × 2 AD patients (each at both baseline and follow-up) and four HVs.

Eq. 2 is used in the reference Logan graphical analysis is given by:

(2) ∫0TROI(t)dtROI(t)=DVR[∫0TREF(t)dt+REF(T)/k¯2ROI(T)]+int⁡'

where ROI(t) and REF(t) are the radioactivity concentrations in a region of interest and a reference region, respectively. The term int' is the intercept and ${\bar{k}}_{2}$ is the average tissue-to-plasma efflux constant. The slope DVR is obtained from the linear portion of the plot (T > t*), where T and t* are midframe scanning time and equilibrium, respectively. If the ratio ROI(t)/REF(t) is constant, the DVR can be obtained without the use of ${\bar{k}}_{2}$.

#### Signal-to-noise ratio

Signal-to-noise ratio, SNR, measures the strength of the signals association to the quantity noise of the data in either sinogram (raw data) or image domain [13]. In this work, signal is defined as the average pixel value, *S*, and noise as the standard deviation of pixel intensities, *N*, within the same outlined ROI. SNR is calculated using Eq. 3

(3) $$SNR=\frac{S}{N}$$

The following four ROIs of similar sizes representing different parts of the brain were drawn, in both AD patients and HVs: frontal cortex sinister (Fsin), frontal cortex dexter (Fdx), parietal cortex sinister (Psin) and parietal cortex dexter (Pdx). According to the study by Klunk [2] the difference in [^11^C]-PIB retention between AD patients and HVs is largest in these areas. SNR was calculated for all ROIs in images generated by the different methods and the mean results of the SNR were compared and plotted.

#### Discrimination power

Discrimination power (DP) is a measurement of quantification differences between two independent groups. It is defined as the difference between absolute value of the means, $\left|{\bar{m}}_{1}-{\bar{m}}_{2}\right|$, divided by the square root of the average of the squared standard deviations (σ_1 _and σ_2_) [14,15]. DP was calculated using Eq. 4.

(4) $$DP=\frac{\left|{\bar{m}}_{1}-{\bar{m}}_{2}\right|}{sd_{p}}$$

where

$$sd_{p}=\sqrt{(\sigma_{1}^{2}+\sigma_{2}^{2})/2}$$

In this study DPs for both images generated by MVW-PCA and reference Logan were calculated using the same ROIs that were used for calculation of SNR (Fsin, Fdx, Psin and Pdx).

In the current study, Graph Pad Prism v. 4.03 (Graph Pad Software, Inc, San Diego, USA) was used to perform all statistical analysis and graphical illustrations.

## Results

To allow a visual comparison between the images generated using the two methods, the image color scale minimum- and maximum-levels were set to the image minimum and maximum intensity, respectively. The maximum and the minimum were calculated from a ROI positioned on the whole brain in the image.

**Figure 1:**
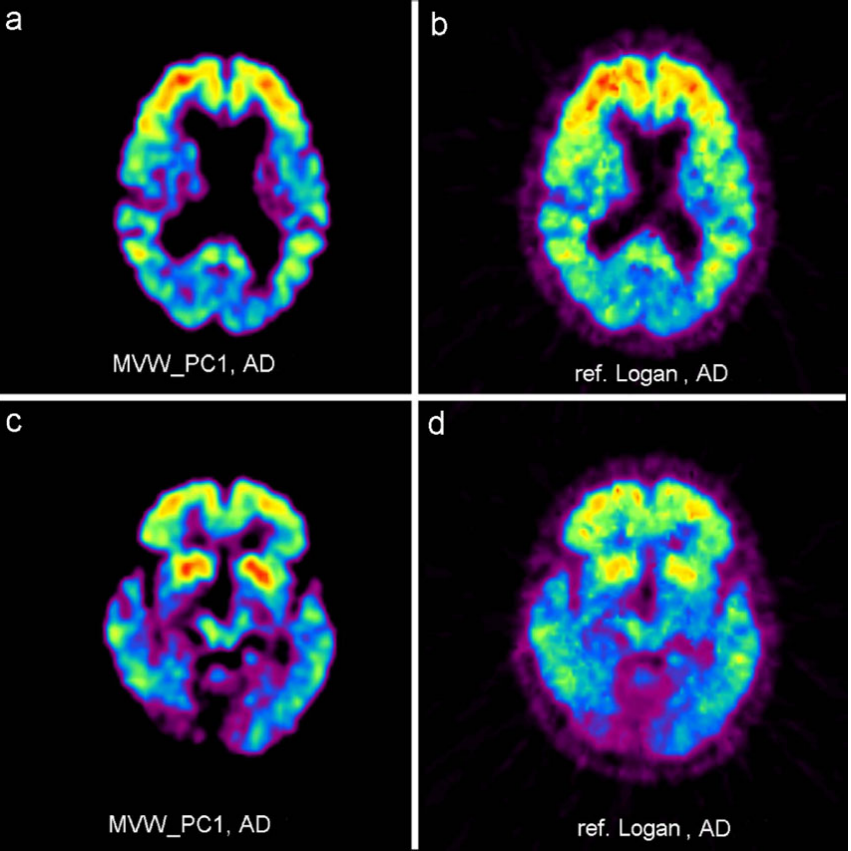
**Visual comparison between MVW-PC1 images generated by application of MVW-PCA on a first scanned (baseline) of an arbitrarily chosen AD patient (a, c) and parametric images generated using reference Logan (b, d) on the same AD patient**.

**Figure 2:**
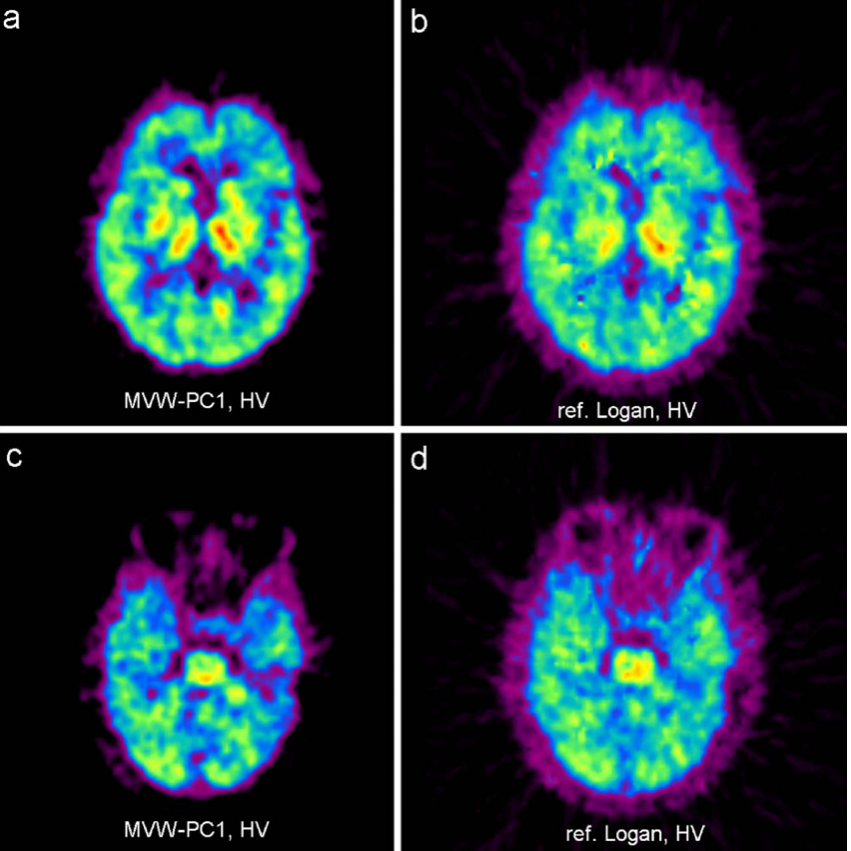
**Visual comparison between MVW-PC1 images generated by application of MVW-PCA (a, c) and parametric images generated using reference Logan (b, d) on a HV**.

The images obtained in each of the four AD patients (both at baseline and follow-up) showed substantial binding of [^11^C]-PIB in the cortical regions of the cerebrum. The binding was highest in the frontal cortex, thus corroborating the finding in most previous [^11^C]-PIB studies [2,4]. The binding in the HVs was very low or virtually absent in the cerebral cortex. No or very little binding of [^11^C]-PIB was seen in the cerebellum of either AD patients or HVs. Fig. 1 and 2 illustrate the comparison of an arbitrary chosen MVW-PC1 image generated by applying MVW-PCA and reference Logan modeling on AD patients and HVs, respectively. The MVW-PC1 images correspond to the kinetics of the tracer in cortical regions such as frontal and parietal cortex, thus representing regions affected by Aβ amyloid deposits in the AD patient. In the HVs, the MVW-PC1 images showed some binding in the thalamus and in basal ganglia.

**Figure 3:**
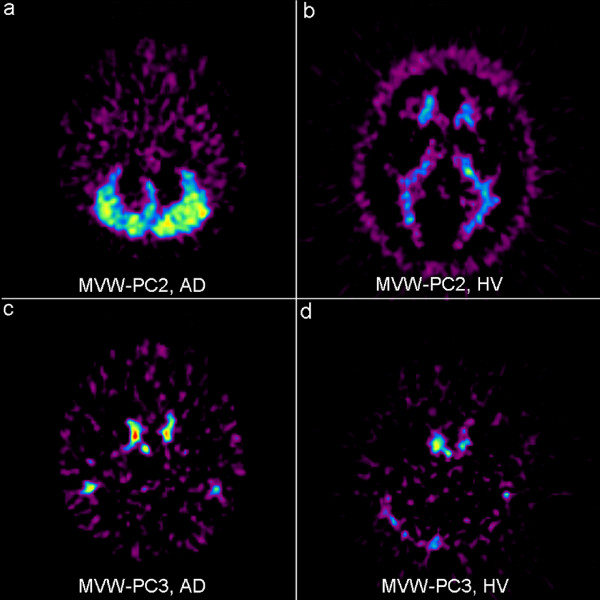
**MVW-PC2 and MVW-PC3 images of same AD patient (a, c) and same HV (b, d) as illustrated in Fig 1 and 2, respectively**.

In the AD patients, the MVW-PC2 images showed cerebellar cortex (Fig. 3a) which is devoid of amyloid, whereas it showed [^11^C]-PIB retention in white matter in the HVs (Fig. 3b). Finally, the MVW-PC3 images contained information of blood flow showing the kinetic behavior of the tracer in blood (Fig. 3c and 3d).

**Figure 4:**
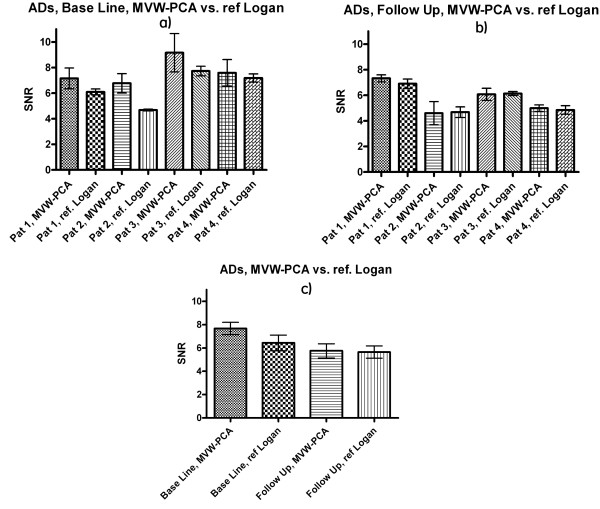
**SNR calculated using MVW-PC1 images generated by applying MVW-PCA and parametric images generated by reference Logan on baseline, follow-up AD patients (a, b) and overall SNR (c)**. The bars show mean ± standard error of the mean (s.e.m.)

**Figure 5:**
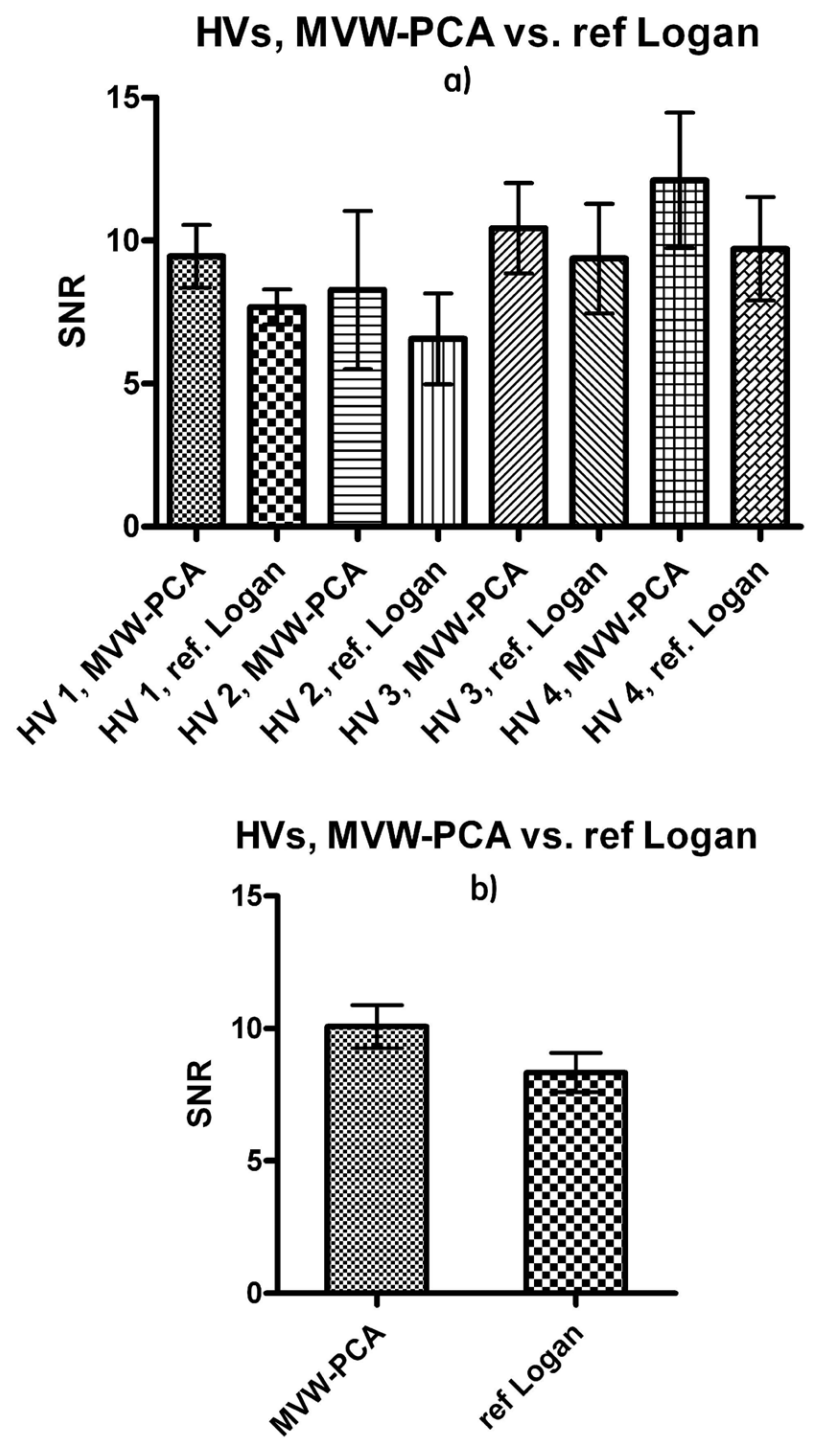
**SNR (a) and overall SNR (b) are calculated using MVW-PC1 images generated by applying MVW-PCA and parametric image generated by reference Logan on HVs**. The bars show mean ± s.e.m.

The SNRs were calculated for the ROIs in images generated using MVW-PCA and reference Logan. The SNR was slightly improved and higher in the MVW-PC1 images than in the reference Logan images in both AD patients (Fig. 4) and HVs (Fig. 5). The differences were significant in baseline study in AD patients (p = 0.039) and in HVs (p < 0.01) (Table 1).

**Table 1:** Table 1 Statistical comparison between different areas in AD patients ( both in baseline and follow up) and HVs when using different methods.

Paired t-test, two-tailed	P value	Mean of differences
SNR, MVW-PCA vs. Ref. Logan, base line	0.0389	1.245

SNR, MVW-PCA vs. Ref. Logan, follow-up	0.4462	0.1021

SNR, MVW-PCA vs. Ref. Logan, HVs	0.0078	1.734

Results obtained performing paired t-test between the ROIs from the frontal and parietal cortices (Fsin, Fdx, Psin and Pdx) in images generated using MVW-PCA and reference Logan.

**Figure 6:**
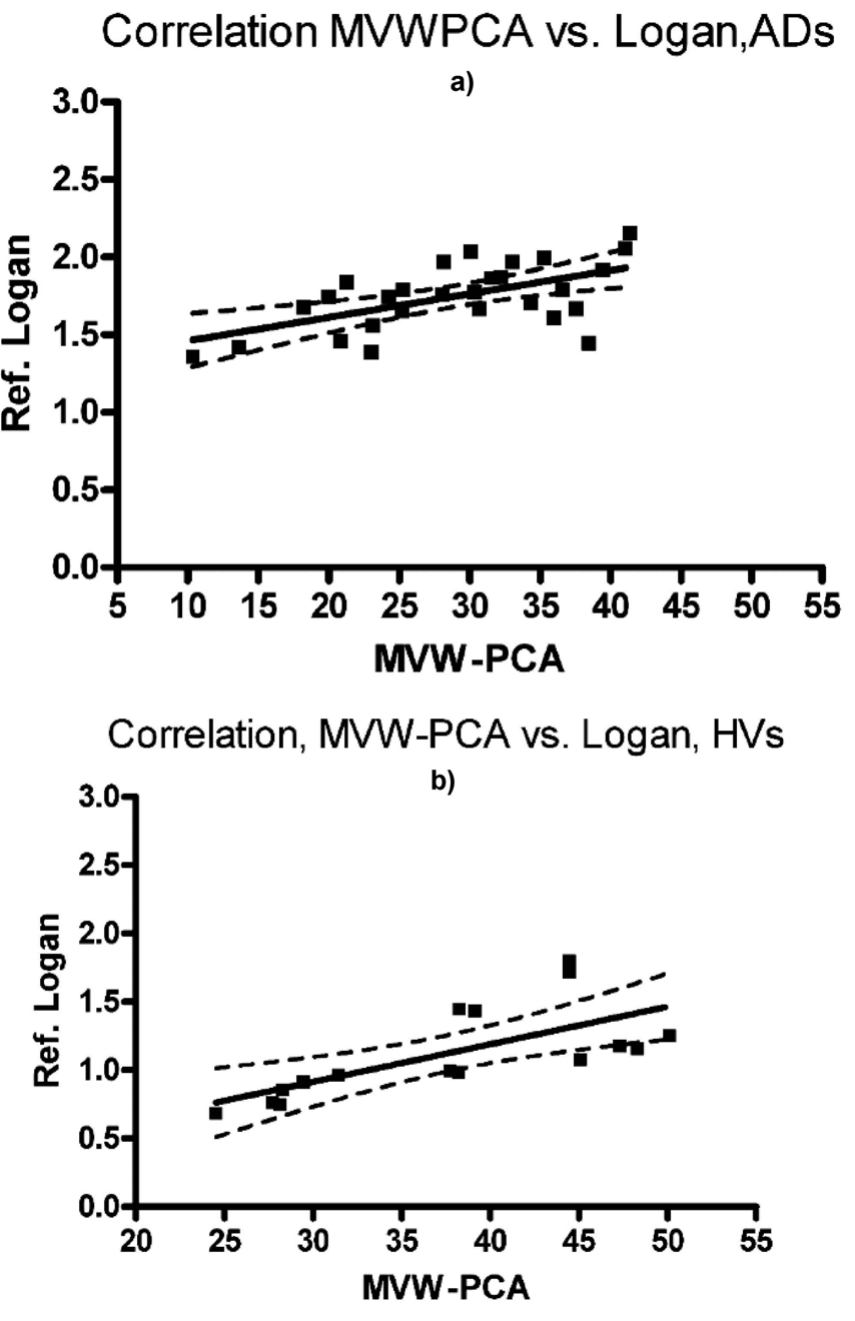
**a Linear regression showing correlation between MVW-PCA and parametric image generated using reference Logan with respect to signal (mean) in AD patients**. ROIs representing four cortical regions (Fsin, Fdx, Psin and Pdx) were used for comparison. Slope = 0.015 ± 0.004 and intercept = 1.310 ± 0.124. b. Linear regression showing correlation between MVW-PCA and parametric image generated using reference Logan with respect to signal (mean) in HVs. ROIs representing four cortical regions (Fsin, Fdx, Psin and Pdx) were used for comparison. Slope = 0.028 ± 0.008 and intercept = 0.086 ± 0.292.

Fig. 6a and 6b illustrate the correlation (Spearman correlation method) between MVW-PC1 and reference Logan. There was a significant correlation between the two methods in both AD patients (both baseline and follow-up) and HVs with correlation coefficients of 0.61 and 0.80, respectively.

**Figure 7:**
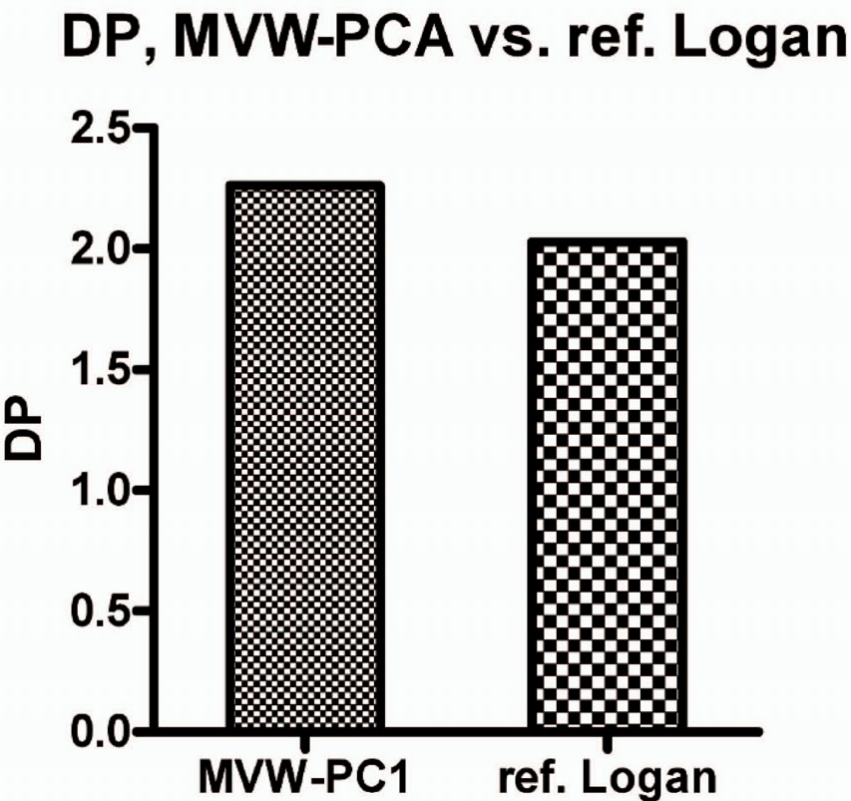
**DP comparison between images generated using MVW-PC1 and reference Logan in which quantitative ROI-values from the four cortical regions between the AD patients (baseline and after follow-up) and the HVs were used**.

Fig. 7 illustrates the comparison between MVW-PC1 and parametric images generated using reference Logan with respect to DP in this study. The quantitative ROI-values from the four cortical regions between the AD patients (baseline and follow-up) and the HVs were used in the calculation of DP. The DP was slightly higher in the MVW-PC1 image as compared to the parametric images obtained using reference Logan.

## Discussion

Kinetic modeling can be used to estimate physiological parameters in vivo and to analyze dynamic images in human PET studies using various tracers. However, prior knowledge about kinetic behavior of the tracer under study is needed to select an appropriate modeling method to analyze the data. For instance, it is necessary to know whether the administered tracer binds reversibly or irreversibly to the target system and whether there is any proper reference region devoid of specific binding of administered tracer (if reference region is used as input function). To overcome these drawbacks we have previously introduced a new approach for application of PCA, and more specifically MVW-PCA, on dynamic PET images [5,6]. The images generated using MVW-PCA, were shown to contain detailed high quality anatomical information. One important feature of MVW-PCA, as well as of other kinetic modeling, is the possibility to separate regions in different compartments, based on their different kinetic properties [5,6].

The aim of the present study was to explore whether results obtained using MVW-PCA give similar results as using a kinetic modeling method such as reference Logan. We studied the detection and visualization of regional differences in [^11^C]-PIB retention between affected and unaffected regions in the human brain in patients with AD as compared with HVs. Moreover, we also compared the images generated using MVW-PCA and reference Logan with respect to signal extraction based on calculating SNR and differentiation between AD patients and HVs using DP.

Results obtained from this work show that MVW-PCA generates images which demonstrate similar regional changes in kinetic behavior of administered tracer in human brain in both AD patients and HVs as images generated using reference Logan. Moreover, several MVW-PC images are generated when applying MVW-PCA, images that provide additional information by discriminating and visualizing regions with different types of kinetics in the different MVW-PCs. It is thus possible to obtain separate images of e.g. regions with specific binding vs regions with nonspecific binding or vs. blood compartment. This is a property of great value that is not obtained when using reference Logan. This might improve the understanding of the kinetic behavior of administered tracer by illustrating additional differences in different parts of the brain. The MVW-PCA method is automated; faster and fewer numbers of clinical experiments are required to determine efficiency of the tracer or tracers of interest used in human PET studies. Furthermore, these images show slightly improved SNR and DP compared to the images generated using reference Logan.

So far, MVW-PCA does not yield absolute quantitative binding data, but only relative data. The reference Logan method is however a quantitative method that estimates the distribution volume ratio of the tracer, which is a linear function of receptor availability. MVW-PCA retains the relative proportions between different regions with different amyloid retention with higher qualitative values, but can so far only be used as a qualitative tool. Future studies will be performed to enable quantitative analysis of images generated using MVW-PCA.

## Conclusion

To conclude, the results obtained from this study demonstrate the potential of MVW-PCA as a multivariate method that by using the whole dynamic dataset without modeling assumptions generates high quality images. Generated images using MVW-PCA demonstrate similar regional changes in kinetic behavior of administered tracer as compared with images generated using the reference Logan kinetic modeling method with the cerebellum as a reference region. In addition, MVW-PCA generates images with an enhanced contrast and higher SNR and DP. The present evaluation shows that MVW-PCA can be used in the study of beta amyloid distribution in AD patients as compared to HVs. This might improve the clinical interpretation and diagnosis of the PET studies on the human brain when using newly developed tracers or when using existing tracers in new clinical applications where kinetic behavior of employed tracer is not known. In addition, MVW-PCA could be used as a new technique, applicable not only on dynamic human brain studies but also on dynamic human cardiac studies when using PET.

## Competing interests

The authors declare that they have no competing interests.

## Authors' contributions

Authors PR, AR, HE participated in the design of the study. They created the introduced method, performed the image and data analysis, and drafted the manuscript.

HH and BL contributed to some of the practical approaches and the writing of the paper.

## Pre-publication history

The pre-publication history for this paper can be accessed here:


